# CT Scanning in Identification of Sheep Cystic Echinococcosis

**DOI:** 10.1155/2017/4639202

**Published:** 2017-08-31

**Authors:** Rui Mao, Hongzhi Qi, Lei Pei, Jie Hao, Jian Dong, Tao Jiang, Abudula Ainiwaer, Ge Shang, Lin Xu, Xi Shou, Songan Zhang, Ge Wu, Pengfei Lu, Yongxing Bao, Haitao Li

**Affiliations:** ^1^Department of Radiation Oncology, The First Affiliated Hospital of Xinjiang Medical University, Urumqi 830054, China; ^2^Abdominal Surgery & Oncology Department, Changji Branch of the First Affiliated Hospital of Xinjiang Medical University, Changji, Xinjiang 831100, China; ^3^State Key Laboratory Incubation Base for Xinjiang Major Diseases Research and Xinjiang Key Laboratory of Echinococcosis, The First Affiliated Hospital of Xinjiang Medical University, Urumqi, Xinjiang 830054, China

## Abstract

**Objective:**

We aim to determine the efficiency of CT in identification of cystic echinococcosis in sheep.

**Methods:**

Fifty-three sheep with liver cysts confirmed by ultrasonography were subject to CT scan to evaluate the number, size, and type of the cysts in liver and lung, confirmed using necropsy. The correlation of numbers between liver cysts and lung cysts was calculated using Pearson analysis.

**Results:**

Necropsy indicated a 98% consensus on size, location, number, and activity compared with CT scan. The viable cysts were 53.1% and 50.6% in the liver and lung, respectively. Among the cysts in liver, 35.5%, 9.5%, 5.7%, 10.2%, and 39.1% were Types CE1, CE2, CE3, CE4, and CE5, respectively. The cysts in the lungs, 17.4%, 26.9%, 12.1%, 11.6%, and 32.1%, were Types CE1, CE2, CE3, CE4, and CE5, respectively. A significant correlation was noticed between the number of cysts in liver and those in lung (*R* = 0.770, *P* < 0.001).

**Conclusions:**

CT scan is a suitable tool in determining the size and type of cystic hydatid cysts in both liver and lung of sheep. A significant correlation was noticed between the numbers in liver and lung, indicating that lung infection was likely due to the expansion of liver cyst burden pressure.

## 1. Introduction

Cystic echinococcosis (CE), a complex and chronic disease caused by the larvae of the* Echinococcus granulosus*, is a parasitic zoonosis affecting the public health and animal production worldwide, especially in the Mediterranean region, Central Asia, South America, and North Africa [1, 2]. Liver is one of the most commonly affected organs in CE patients [3]. Besides surgery, chemotherapy using albendazole (ABZ) has been commonly used for treating CE. However, its efficiency may be overestimated as ABZ only resulted in an apparent cure in up to 30% of cases [4–6]. Besides, cyst degeneration occurs in up to 20% of patients [7]. Therefore, it is urgent to develop new drugs for treating CE.

Sheep are the most adapted intermediate hosts to the parasite and are suitable for the study focused on the hydatid disease. However, there are no studies focusing on image techniques to identify hydatid cysts in sheep. To precisely identify and measure the cyst size and status, we used CT to determine the size and status of cysts in liver and lung of sheep naturally infected with* E. granulosus*. Currently, ultrasonography has been preferred for the diagnosis of CE in clinical practices [8, 9]. Besides, cystic typing has been established using the ultrasound techniques according to the WHO guidelines [10]. Nowadays, no typing has been proposed based on the CT scan despite the fact that CT is superior to ultrasound in the accurate localization and high resolution [11]. Compared with the CT scan, the resolution of ultrasonography was lower, which could not display the slight calcification in the cysts. In contrast, CT contributes to the evaluation of CE activity* in vivo*, and such technique has been commonly used for the preoperative evaluation and postoperative outcome analysis [12]. In this study, based on the images of the cysts determined and according to ultrasonography classification of hydatid cysts, we classified the hydatid cysts existing in liver and lung of the sheep using CT scan.

## 2. Materials and Methods

### 2.1. Animals

Fifty-three female sheep from Bayinbuluke Town, a seminomadic community in Hejing County of Xinjiang, were used in this study. Initially, the sheep were screened by ultrasonography and animals with at least 1 active cyst and were selected for the subsequent CT scan test. The study protocols were approved by the Animal Ethic Committee of the First Affiliated Hospital of Xinjiang Medical University.

### 2.2. CT Scan

Before CT scan, the animals were anesthetised by a qualified veterinarian from the Animal Centre of the First Affiliated Hospital of Xinjiang Medical University using standard regimen. Sheep were initially subject to administration of atropine sulfate (0.1 mg/kg) via subcutaneous injection, followed by intramuscular injection of Zoletil (5 mg/10 kg, VIRBAC laboratories, 06516 Carros-France) 15 min later. Subsequently, 10 mL mixture of xylazine hydrochloride, diazepam, atropine, and normal saline (2 : 2 : 1 : 5) was given via intravenous injection (1 ml/10 kg). After anaesthesia, the sheep were transferred to CT room and laid on the CT examination table in a supine position. The animals were wrapped up using a thermoplastic film around the abdomen for the fixation. Thoracic and abdominal CT was performed using the PHILIPS Brilliance BIG Bore CT scanner. The slice thickness was 5 mm, and the space between slices was 5 mm. All sheep were subject to necropsy after CT scan.

CT images were analyzed using the workstation to evaluate the size of cysts. The number of cysts in liver and lung was calculated, and the cysts were classified according to the criteria issued by World Health Organization-Informal Working Group on Echinococcosis [13, 14], including CE1 (unilocular cyst), CE2 (daughter cyst), CE3 (collapsed internal membrane type), CE4 (consolidation type), and CE5 (calcification type). The organ infection intensity was calculated based on the ratio of number of cysts in the infected organ to the number of sheep with positive organ infection. All cysts were observed under a microscopy, and echinococcal cysts were confirmed by the presence of protoscoleces and laminated layers.

### 2.3. Statistical Analysis

SPSS 22.0 was used for statistical analysis. Pearson correlation analysis was used to investigate the correlation between the numbers of hepatic and pulmonary cysts. *P* < 0.05 was considered to be statistically significant.

## 3. Results

### 3.1. Criteria for Classification of Echinococcal Cysts in Sheep

**Figure 1:**
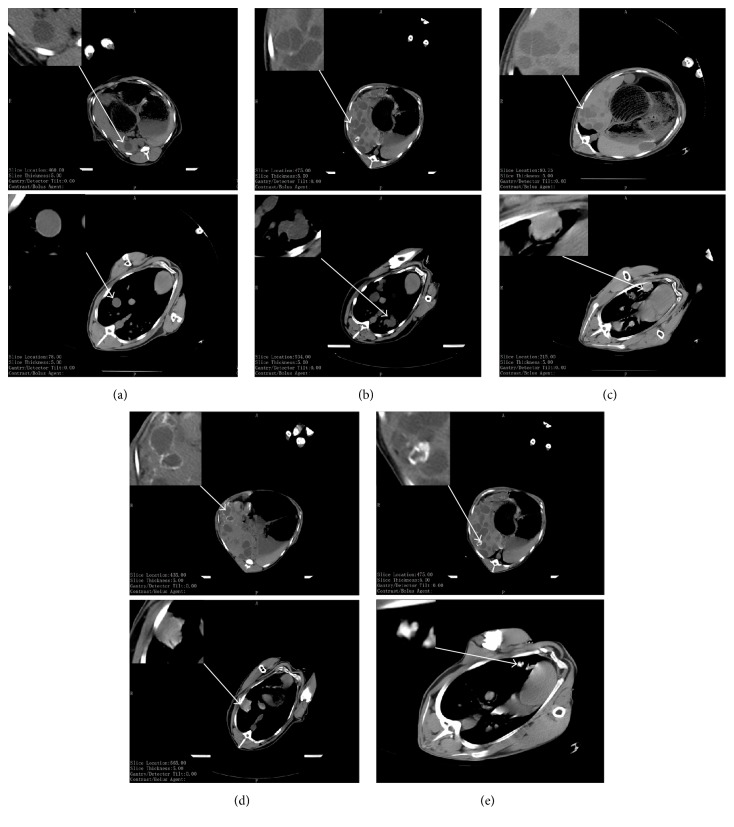
Imaging features of cystic echinococcocal cysts detected by CT. (a) CE1 was in a normally round or oval shape, with a size range of 1–10 cm. (b) Typical image of CE2 with “cyst in cyst,” “honeycomb-like,” or “rose petal” structures. (c) A typical image of CE3 with hydatid cyst wall turning thick and rough. The density of the cyst fluid increased and even showed a pot of calcification. (d) A typical image of CE4 with calcification in the partial cysts. (e) Image of CE5 with a thick calcified wall in the cyst.

Classification criteria for* E. granulosus *cysts were showed in Figure 1, which were mostly based on the classification by WHO image working group 2000. CE cysts in sheep liver (or lung) were often multiple cysts in different sizes.

### 3.2. Calculation of the Cyst Number in CT Images

**Figure 2:**
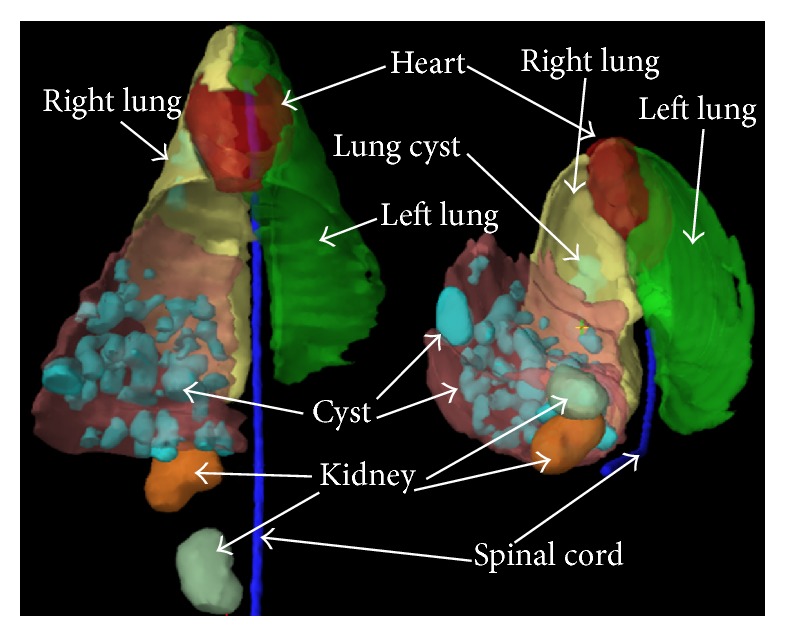
Three-dimensional CT reconstruction of lung and liver in sheep infected with* E. granulosus*.

Presence of cysts in both liver and lung was observed in 42 sheep according to the plain CT scan. Eleven sheep showed presence of cysts only in liver. Many sheep showed multiple cysts in liver (Figure 2), with the maximal number of 134 cysts in 1 sheep. A total of 906 cysts were identified by the CT scan, among which 716 (79.0%) were located in the liver while the rest 190 (21.0%) were in the lung (Table 1). The average cyst number in liver was significantly higher than that in lung (15.85 versus 5.10, *P* < 0.01, Table 1).

### 3.3. Classification of Hepatic and Pulmonary CE Cysts

To assess the cyst status, we used the WHO ultrasound classification criteria [11] to classify the cysts in the liver and lung using the CT images.  Table 2 showed the classification and distribution of the cysts in liver and lung.

**Figure 3:**
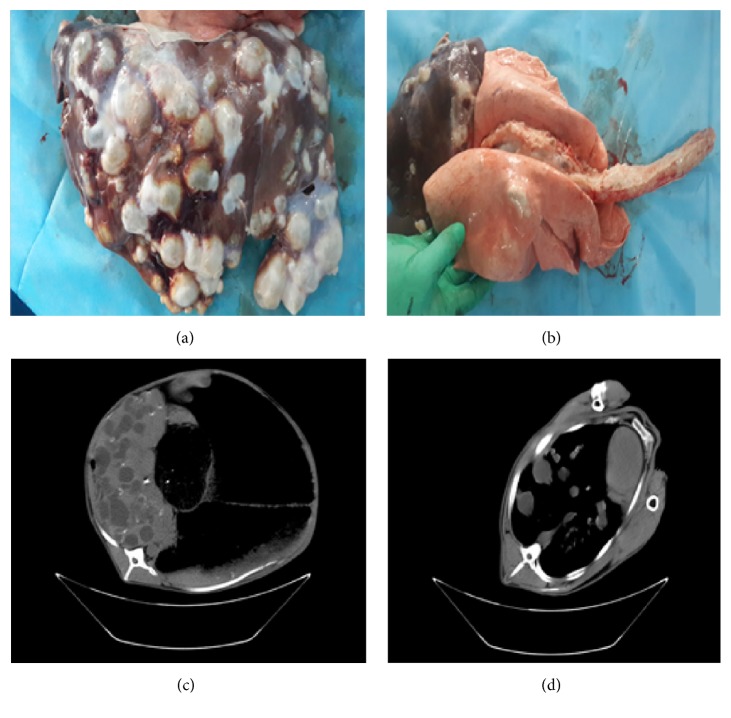
Autopsy examination indicated sheep infected* Echinococcus granulosus* cysts and CT scan of these cysts (before slaughter). Autopsy examination of CE cysts in sheep liver (a) and lung (b). Plain CT scan identifying CE cysts in a sheep liver (c) and lung (d).

Presence of protoscoleces was observed in 53.1% of cysts in liver, while 46.9% showed presence of protoscoleces in the cysts in lung (Figure 3). For the liver cyst, 21.4% of the cysts were categorized into CE1, while the others were categorized into CE2 (25.2%), CE3 (4.9%), CE4 (15.2%), and CE5 (33.3%), respectively. For the lung cysts, 26.5% of the cysts were categorized into CE1, while the others were categorized into CE2 (24.0%), CE3 (0.7%), CE4 (0.3%), and CE5 (28.5%), respectively.

### 3.4. Correlation of Liver Cysts with Lung Cysts

We used Pearson correlation analysis to determine the correlation of cysts between liver and lung. Our data showed a significant positive correlation (*R* = 0.770, *P* < 0.01). The correlation was confirmed using linear regression. The hepatic CE cyst number served as the independent variable *χ* and the pulmonary CE cyst number served as the dependent variable *y*. Our data revealed a positive correlation between the two parameters (*R* = 0.661, *R*^2^ = 0.437, *F* = 26.367, *P* < 0.001). The correlation indicated that the pulmonary CE cyst number increased with the increase of cysts in the livers of the sheep.

## 4. Discussion

This is the first report using CT scan to characterize the features of CE in sheep. The same parameters similar to those used for clinical diagnosis and follow-up were used in this study, which approved that all these parameters were suitable for scanning. In addition, as proposed in human beings, we confirmed that the criteria described for the international classification of CE cysts can be used for the classification of cysts observed after CT scan.

The most significant feature of sheep infected with CE is that the cysts appear in multiple cysts, which may be at various stages. The necrotic cysts in various stages of cysts indicated the nature infection history or host immune attacking consequences. The necrosis of CE cyst is important for understanding biology and drug and vaccine development. Currently, ultrasonography has been routinely used for clinical diagnosis, characterization, and measurement of hydatid cyst in the livers of CE patients [15]. Previously, such technique has also been used in sheep to assess* E. granulosus* infection in a flock [16]. However, the stage and status cannot be precisely evaluated due to the body shape of sheep and the usual field conditions of examination. In addition, the number of cysts in a single sheep is usually higher than that in a human individual, which limits a precise quantitative evaluation of the cysts and the location in liver [17]. Finally, the utilization of ultrasonography for detecting and characterizing the lung cysts is very limited in humans and sheep. As an alternative method in the scanning of cysts in lung, we determined the number and type of the cysts in lung and liver using CT scan in this study. Our data showed that the CE structures could be clearly displayed by CT scan, especially for detecting the calcification in cysts. Additionally, the size and profile showed no statistical differences in the CE cysts in liver and lung detected using CT and ultrasonography. Unlike the ultrasonography with lower resolution on the cysts and availability of small calcified lesions, CT can be used for scanning the whole body and obtain accurate size and shape of cysts. Therefore, it has been commonly used for the evaluation of treatment outcome of CE in clinical practice.

In human CE cases, about 70% of cysts were in liver, and about 20% of cysts were in lung with the rest in heart, kidney, and other different organs [18]. Presence of infection in lung and liver was reported in about 1% of human cases simultaneously. However, in the 53 sheep with liver cysts, nearly 80% of the sheep had lung cysts. Correlation analysis showed a positive correlation between the pulmonary cyst number and the hepatic cyst number. With the increase of the CE cysts in liver, pulmonary cyst infection number also increased, and significant correlation was noticed between the cyst numbers. This relationship suggested that hepatic CE infection intensity is an important factor affecting pulmonary CE infection intensity. Meanwhile, the present study also indicated that no lung infection was detected in the high hepatic infection intensity group. On this basis, we implied that hepatic blood vessel blocking function was related to differences among individuals.

In summary, we firstly perform CT scan to determine the number of CE cysts in sheep liver and lung. Our data show that CT scan is a useful tool for detecting cyst size and shape and even the status of cyst.

## Figures and Tables

**Table 1:** Characterization of CE infection by computed tomography in 53 sheep with cysts disclosed at ultrasound examination.

	Liver	Lung
Number of infected sheep	53	42
Total number of cysts	840	214
Infection rate (%)	100	79.20
Infection intensity	15.85	5.1
Cyst diameter range (mm)	1~36	1~28
Average diameter (mm)	26.21	20.37

**Table 2:** Comparison of the classification of hepatic and pulmonary CE cysts.

	Liver	Lung
CE1	110 (21.4%)	33 (26.5%)
CE2	68 (25.2%)	17 (24%)
CE3	41 (4.9%)	23 (10.7%)
CE4	73 (15.2%)	22 (10.3%)
CE5	280 (33.3%)	61 (28.5%)

## Abbreviations

CT: Computed tomography
CE: Cystic echinococcosis
AE: Alveolar echinococcosis
WHO-IWGE: World Health Organization-Informal Working Group on Echinococcosis
3D reconstruction: Three-dimensional reconstruction.
